# Euglycemic Diabetic Ketoacidosis, Recurrent Genital Abscess, and Proximal Renal Tubular Acidosis With Concurrent SGLT-2 Inhibitor: More Than an Association

**DOI:** 10.7759/cureus.67481

**Published:** 2024-08-22

**Authors:** Anwar Alshaakh Moh'd Mari, Cindy C Iwuagwu, Mark Johnson

**Affiliations:** 1 Internal Medicine, University of Central Florida College of Medicine, Orlando, USA

**Keywords:** adverse drug reaction, proximal renal tubular acidosis (rta), recurrent genital abscesses, euglycemic diabetic ketoacidosis, sglt-2 inhibitors

## Abstract

Sodium-glucose transport protein 2 (SGLT2) inhibitors are a class of antidiabetic medications that have tremendous benefits in diabetic patients through reducing renal tubular glucose reabsorption, therefore inducing a rapid increase in urinary glucose excretion, thus reducing the overall serum blood glucose. However, the medication's use has commonly been associated with emerging complications such as euglycemic diabetic ketoacidosis (eDKA), a rare and life-threatening metabolic disturbance. Other complications that have been associated with this class of medications are recurrent genital abscesses and renal tubular acidosis, which have both been less reported and explored. Below, we detail the case of a woman who was on empagliflozin, an SGLT2 inhibitor, for only two months and developed life-threatening eDKA, recurrent genital abscesses, and proximal renal tubular acidosis all within the two months of initiation of the medication.

## Introduction

Euglycemic diabetic ketoacidosis (eDKA) is a rare yet potentially life-threatening metabolic complication observed in individuals with diabetes mellitus (DM) who are treated with sodium-glucose co-transporter inhibitors (SGLT-2i) [1]. While the association between SGLT-2i and eDKA has been extensively reported, the occurrence of recurrent genital abscesses in patients concurrently taking these medications remains a less explored phenomenon. The reported incidence of SGLT-2i-induced DKA is 0.16-0.76 events per 1000 patients per year [2]. In addition, proximal renal tubular acidosis (RTA) as a result of SGLT2-i is considered to be relatively rare and lacks extensive documentation. In this report, we are showcasing an exceptional instance where all of these complications co-occurred after the recent initiation of SGLT-2i.

## Case presentation

**Figure 1:**
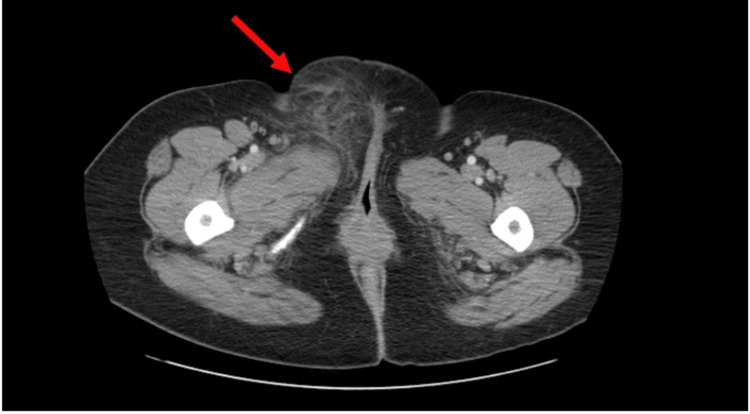
Computerized tomography (CT) of the abdomen and pelvis reveals extensive edema in the right groin and right vulva with a 1.2 cm early abscess.

A 42-year-old woman with a history of non-insulin-dependent diabetes, intolerant of metformin, who was started on Empagliflozin two months previously, presented to the emergency department with symptoms of redness, swelling, and pain in the right vulvar area with associated nausea, one episode of vomiting, fever, chills, and a lack of appetite. The patient denied any diarrhea. Upon admission, the physical examination indicated the presence of severe sepsis with tachycardia at 120 bpm, tachypnea at 26 breaths per minute, and a mild fever of 100.4 F. There was significant tenderness to light palpation of the entire right lower abdominal quadrant. There was evident erythema, swelling, and tenderness in the right vulvar region, extending to the mons pubis area, which was approximately 7.0 x 7.0 x 7.0 cm in size, with some extension into the right lower abdominal quadrant. No purulent discharge or fluctuant mass was noted on the exam. Laboratory testing supported the sepsis identification with leukocytosis of 14,000/mm (reference range 5000-10,000/mm). Further laboratory investigation also revealed a high anion gap metabolic acidosis with compensatory respiratory alkalosis with an anion gap of 24mEq/L (reference range 8-12mEq/L), pH 7.07 (reference range 7.35 - 7.45), and bicarbonate 15 mEq/L (reference range 21-36 mEq/L). Arterial pCO2 and HCO3 were 11 mmHg and 10.6 mEq/L (reference range 35 to 45 mmHg and 22-26 mEq/L respectively) (Table 1). Lactic acid in this patient was normal at 1.0 mmol/L (reference range < 2 mmol/L), on arrival. Plasma glucose was 222 mg/dL. Urinalysis was remarkable for 80mg/dl of ketones, 100mg/dl of protein, and >500mg/dl of glucose (Table 2). Beta-hydroxybutyrate was ordered and was found to be 60.8mg/dl (reference range <3 mg/dL), confirming the suspicion of euglycemic diabetic ketoacidosis (Table 3). Hemoglobin A1C (HbA1C) on admission was 8.7%. Further imaging with computerized tomography (CT) abdomen and pelvis revealed extensive edema in the right groin and right vulva with a 1.2 cm early abscess (Figure 1). The patient also reported one other occurrence of perianal abscess since the initiation of empagliflozin. The first episode was described as a large pustule in the left medial thigh that was closely monitored by her primary care for two months without progression to abscess formation or hospitalization. The pustule eventually resolved with a daily warm compress. The patient reports that the current presentation was much more painful, red, and swollen in comparison to the first.

**Table 1:** Serum laboratory findings cells/mm^3^ = cells per cubic millimeter, g/dl = grams per deciliter, % = percentage of RBC in blood, mEq/L = milliequivalents per liter, mmol/L = millimoles per liter, mg/dL = milligrams per deciliter, ml/min = milliliters per minute

	Our patients’ laboratory values	Reference range
WBC (cells/mm^3^)	14,300	4,000-10,500
Hemoglobin (g/dl)	13.8	11.2-15.7
Hematocrit (%)	40.2	34.1-44.9
Platelets Count (cells/mm^3^)	198,000	150-400
Sodium (mEq/L)	136	136-145
Potassium (mEq/L)	3.2	3.5-5.1
Chloride (mEq/L)	100	96-106
Carbon dioxide (mmol/L)	15	21-32
Anion gap	24.2	<10
BUN (mg/dL)	8	7-18
Creatinine (mg/dL)	0.78	0.6-1.3
Estimated GFR (mL/min)	>60	>60
Glucose (mg/dL)	222	74-110
Beta-hydroxybutyrate (mg/dL)	60.8	<3
Lactic Acid (mmol/L)	1.0	<2

**Table 2:** Urine laboratory studies mg/dL: milligrams per deciliter, rbc/HPF: red blood cell per high power field, wbc/HPF: white blood cell per high power field, epi/HPF: squamous epithelial cells per high power field

	Our patient's Urinalysis values	Reference range
Urine color	Yellow	Yellow
Urine appearance	Clear	Clear
Urine pH	5.0	5-9
Urine Specific gravity	1.023	1.005-1.030
Urine protein	100 mg/dL	Negative
Urine glucose	>500 mg/dL	Negative
Urine Ketones	80 mg/dL	Negative
Urine blood	Negative	Negative
Urine nitrite	Negative	Negative
Urine bilirubin	Negative	Negative
Urine urobilinogen	Negative	Negative
Urine leukocyte esterase	Negative	Negative
Urine RBC	0-5 rbc/HPF	0 - 5
Urine WBC	0-5 wbc/hpf	0 - 5
Urine squamous epithelium	0-5 epi/HPF	0 - 5
Urine bacteria	Rare	None seen
Urine mucus	Rare	Rare

**Table 3:** Arterial blood gas results mmHg: Millimeter of mercury, mmol/L: Millimoles per liter

	Our patients ABG values	Reference range
ABG pH	7.31	(7.35-7.45)
ABG pCO_2 _(mmHg)	21	(35-48)
ABG pO_2 _(mmHg)	81.5	(83-108)
ABG pO2/FiO_2_ Ratio (mmHg)	388.1	(>200)
ABG HCO_3 _(mmol/L)	10.6	(21-28)
ABG O_2_ Saturation (%)	95.2	(94-98)
ABG Base Excess (mmol/L)	-13.4	(-2-3)
O_2_ Delivery	Room air	

The patient was admitted to the intensive care unit with a diagnosis of eDKA and treated with IV fluids, insulin bolus, drip, and potassium replacement until the resolution of eDKA. The patient underwent surgical incision and drainage as she was found to have a right suprapubic abscess and a left gluteal abscess. She was treated with empirical antibiotics such as Piperacillin-Tazobactam, Clindamycin, and Vancomycin to cover for any potential necrotizing infections. Wound cultures of abscesses eventually grew Methicillin-sensitive Staphylococcus aureus. The transition was made to Nafcillin based on cultural sensitivities. Following the resolution of her eDKA, the patient developed resistant hypokalemia ranging between 2-3 mEq/L (reference range 3.5-5.2 mEq/L) associated with a persistent non-anion gap acidosis with bicarbonate ranging between 10 and 12 mmol/L. Further evaluation revealed urine pH<5.5 (reference range 4.5 to 7.8), persistent hyperchloremia ranging 110-115 mEq/L (reference range 96-106 mEq/L), and urine anion gap elevation at 16 mEq/L (reference range < 10 mEq/L), all consistent with proximal renal tubular acidosis (RTA). The patient received bicarbonate supplementation along with potassium replacement therapy, which improved her electrolyte imbalances. The patient was discharged with bicarbonate 650 mg once per day along with potassium supplementation, cephalexin 500 mg four times per day for 14 days with continuation of probiotics, discontinuation of empagliflozin, and initiation of insulin NPH-Regular 70/30 and clear instructions for dressing changes. On a six-month follow-up, the patient reported no recurrence of abscesses or pustules and denied any recent hospitalization related to RTA and/or eDKA.

## Discussion

This case highlights the importance of prompt recognition and management of complications in these patients. It also demonstrates the need for a comprehensive approach to address multiple concurrent issues, such as sepsis, eDKA, vulvar abscess, and proximal RTA.

The emergence of persistent hypokalemia with non-anion gap acidosis in this case raises concerns about the possibility of significant complications, such as RTA. Proximal RTA is a type of renal tubular acidosis characterized by impaired reabsorption of bicarbonate in the proximal tubules of the kidneys, leading to metabolic acidosis [3]. Our patient's urine anion gap was higher than the reference range at 16 mEq/L (reference range < 10 mEq/L), confirming our diagnosis. The common triggers of non-anion gap acidosis include diarrhea, recent use of topiramate or diuretics, and administration of normal saline. Each of these was not present in our patient’s history or management. Our patient received lactated ringers exclusively throughout her hospital stay. These findings narrowed the diagnosis to a link between the use of an SGLT2 inhibitor and the development of non-anion gap acidosis related to RTA. Consequently, the SGLT2 inhibitor becomes the leading candidate for diagnosis.

The understanding of how SGLT2 inhibitors can lead to RTA remains unclear; however, various theories exist regarding its mode of action. As we know, SGLT2i targets the SGLT2 cotransporters on the apical membrane of the proximal convoluted tubule, inhibiting the reabsorption of glucose [4]. Based on Onishi, Akira, et al. (2020) [5], it has been suggested that the activities of SGLT2 and Na+-H+ exchanger 3 (NHE3) are interconnected. Consequently, the suppression of NHE3 may play a role in the natriuretic response observed with SGLT2 inhibitors. The increased urinary excretion of Na+ and bicarbonate causes an increased urinary pH in wild-type mice, leading to serum metabolic acidosis. The study was done on SGLT2 inhibition by empagliflozin. Further research is required to gain a comprehensive understanding of these mechanisms.

The mechanism underlying the development of eDKA with SGLT-2i is believed to involve increased ketogenesis, reduced insulin secretion, and decreased insulin activity [1].

Of note, some potential side effects associated with SGLT-2i may include an increased risk of genital bacterial and/or mycotic infections in both men and women. It is also widely acknowledged that individuals with DM are generally at a higher risk of developing abscesses, including genital abscesses. The occurrence of recurrent abscesses after starting SGLT-2i treatment in this case strongly suggests a potential association with the medication, leading to the consideration of discontinuing its use. Six months after discontinuing empagliflozin, our patient reported no recurrence of eDKA and denied any recurrent abscesses, which confirms that the initiation of empagliflozin likely triggered these life-threatening outcomes.

## Conclusions

SGLT-2i is generally a safe and beneficial medication for reducing blood sugar in diabetic patients. However, like many other medications, adverse effects are risks that need to be considered in this patient population. Although eDKA in the setting of well-controlled diabetes by SGLT-2i is uncommon, this case serves as a reminder that it should be a differential diagnosis in patients presenting with similar symptoms. It is also important to strongly consider RTA when assessing metabolic abnormalities and consider genital bacterial or mycotic infections in patients presenting with sepsis in the setting of newly initiated SGLT-2i.
